# Tachycardia induced cardiomyopathy due to ectopic atrial tachycardia originating from the atrial appendage: A case series and review of literature

**DOI:** 10.1016/j.ipej.2025.03.001

**Published:** 2025-03-06

**Authors:** Abhinav Aggarwal, Anil Yadav, Ankit Jain, Anunay Gupta

**Affiliations:** Department of Cardiology, VMMC & Safdurjung Hospital, India

**Keywords:** Atrial tachycardia, Tachycardia induced cardiomyopathy, Atrial appendage, Ivabradine, Electrophysiology, Radiofrequency ablation

## Abstract

This case series describes four cases of tachycardia-induced cardiomyopathy due to incessant ectopic atrial tachycardias from the atrial appendage (three from the right atrial appendage, one from the left). P wave morphology changes on surface 12-lead electrocardiogram can be used to diagnose this relatively rare subset of tachycardias and localise the site of origin. Tachycardia induced cardiomyopathy is relatively more common in atrial tachycardias from the atrial appendage as compared to tachycardia from other sites^1,2^. Radiofrequency ablation is the treatment of choice and is associated with a high success rate. Oral ivabradine is another treatment option for cases where ablation is unsuccessful or if the patient is unwilling for ablation. For rare cases refractory to other treatment measures, surgical excision of the atrial appendage may be needed.

## 1 Introduction

Ectopic atrial tachycardias (EAT) originating from atrial appendage are more common in younger patients and are more often incessant and associated with left ventricular dysfunction as compared to tachycardias from other sites [1,2]. Identification of this relatively rare subset of supraventricular tachycardia using P wave morphology changes on surface 12-lead electrocardiogram is important, especially because radiofrequency ablation of these tachycardias is associated with a high success rate and a low recurrence rate. Also, successful treatment of these tachycardias is associated with an improvement in left ventricular function in the case of tachycardia-induced cardiomyopathy [1].

## 2 Case 1

**Fig. 1:**
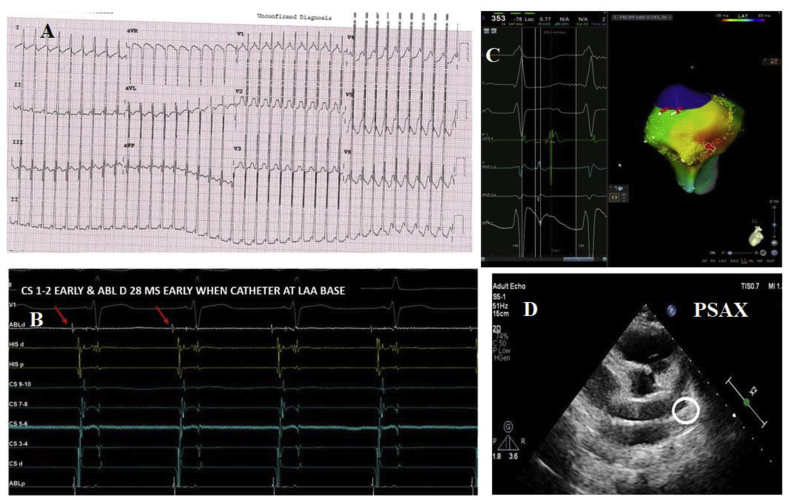
(A)12 lead ECG showing Narrow QRS long RP tachycardia with rate 260/min. (B)EGM showing CS -1,2 earlier than surface ECG P waves and also showing the signals at the ablation catheter. (C)3D EAM showing unipolar and bipolar signals at the site of ablation. (D)Location of Ablation catheter at LAA base: confirmed on TTE.

An 11-year-old male presented with rapid, regular intermittent palpations for 1 year and dyspnoea on exertion for 6 months. His heart rate would vary from 70 to 250/min. 12 lead ECG showed narrow complex long RP tachycardia (Fig. 1A). P wave morphology was suggestive of EAT originating from the left atrial appendage (LAA): upright P waves in V1- V6, which were notched in V1, upright P waves in inferior leads and negative P in 1 & AVL. Transthoracic Echocardiography showed a dilated left ventricle with global hypokinesia (EF 15 %). Tachycardia was refractory to beta blockers and ivabradine.

CARTO 3D EAM (Electroanatomic mapping) system was used for mapping. During tachycardia, the tip of the decapolar catheter in the coronary sinus showed signals earlier than the surface P waves (Fig. 1B). EAM revealed the earliest Signal in the LAA base which was 29 msec earlier than the surface P waves (Fig. 1C). Using an irrigated ablation catheter, ablation was done at the LAA base after confirming the location fluoroscopically & by transthoracic echocardiography (Fig. 1D). Tachycardia terminated within 3 seconds of the ablation, and no recurrence was seen even after atrial burst pacing & isoprenaline. The average power used was 30 Watts with an RF duration of 3 minutes. The total procedure time was 45 minutes, and the fluoroscopy time was 5 minutes. The patient is on routine follow-up for 10 months, is in normal sinus rhythm, and ejection fraction has normalized with no recurrence of tachycardia.

## 3 Case 2

**Fig. 2:**
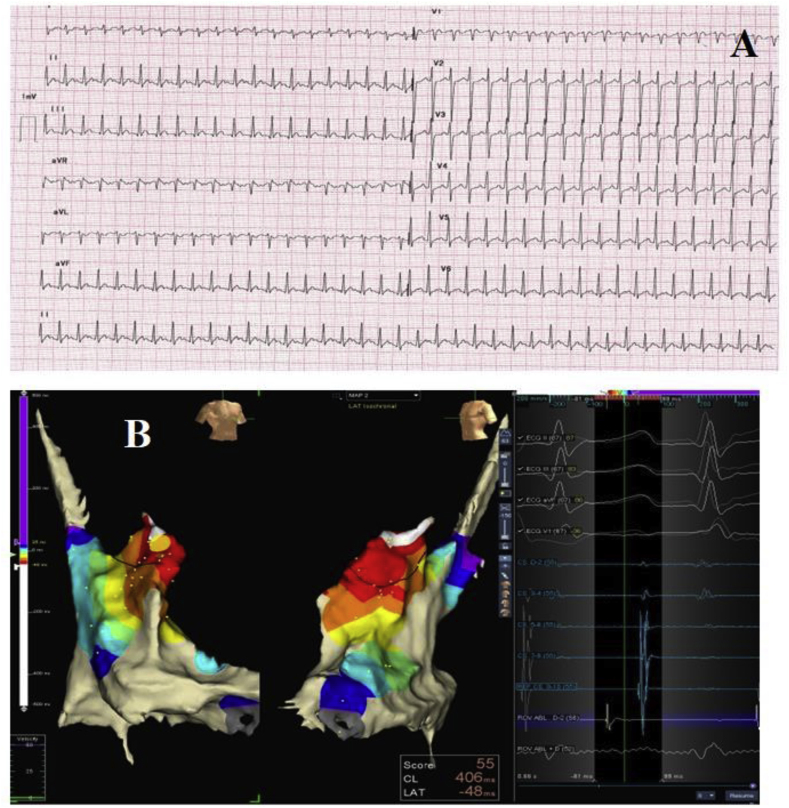
(A) 12 lead ECG showing Narrow QRS long RP tachycardia. (B) Activation Map Revealed Earliest Signal in the right atrial appendage, which was 48 msec earlier than the surface QRS.

A 22-year-old female with no other comorbidities presented to our hospital with complaints of palpitations and dyspnoea on exertion for the past 2 years. Physical examination was unremarkable. Electrocardiography showed narrow complex long RP tachycardia with a ventricular rate of 240 beats per minute (Fig. 2A). On giving adenosine, P waves were clearly visible with upright P waves in leads V1-V6, inferior leads and lead 1. P waves were more negative in aVR than lead aVL (Supplementary Fig. 1), suggestive of EAT originating from the superior part of the right atrium. Transthoracic echocardiography revealed an ejection fraction of 35 % and global LV hypokinesia with no other abnormalities. The patient had incessant tachycardia on cardiac monitoring.

Using NAVX 3D EAM system, an activation map was made using point-by-point mapping, which revealed the earliest signal in the right atrial appendage 48 msec earlier than the surface P waves (Fig. 2B). After localizing the site of origin, using an SL 0 long sheath for support, ablation was done using an irrigation ablation catheter (Supplementary Fig. 2). Tachycardia terminated within 3 seconds of the ablation but reappeared after 5 minutes of waiting. Further mapping revealed the earliest signal at the tip of the right atrial appendage, which was 53 msec ahead, with QS in a unipolar voltage signal. Ablation at that site was successful, with no recurrence, with atrial burst pacing and isoprenaline after 30 minutes of waiting. Wider regions of ablations were given in the RA appendage in the waiting period to decrease the chances of recurrence. The average power used was 30 Watts with an RF duration of 5 minutes. The total procedure time was 85 minutes, and the fluoroscopy time was 6 minutes. The patient is on routine follow-up for 11 months, and ejection fraction has improved over time with no recurrence of symptoms.

## 4 Case 3

**Fig. 3:**
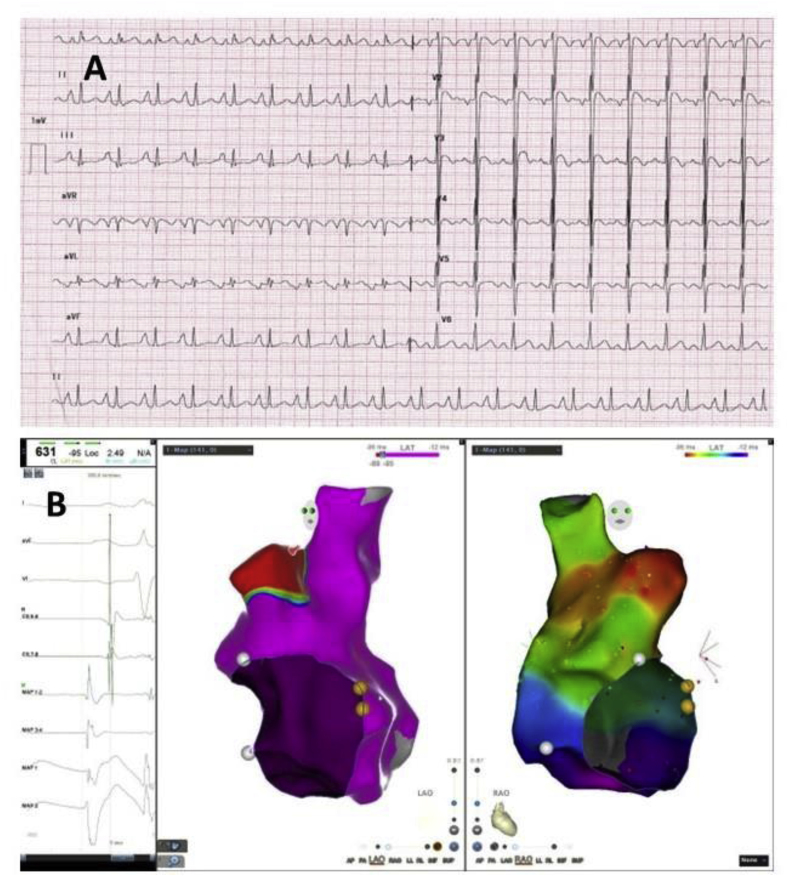
(A) ECG was suggestive of atrial tachycardia with likely origin from right atrial appendage: Negative P waves in V1, V2 with upright P waves in inferior leads. (B) 3D EAM showing Earliest Signal in RAA.

A 22-year-old male, who was a known case of ectopic atrial tachycardia, well controlled on oral ivabradine, presented with recurrent palpitations and signs and symptoms of heart failure after stopping ivabradine. The patient stopped ivabradine on his own despite being counselled about the nature of the disease and the treatment being given, as he was symptom-free. His ECG was suggestive of EAT with likely origin from right atrial appendage: Negative P waves in V1, V2 with upright P waves in inferior leads (Fig. 3A), cardiac monitoring showed incessant tachycardia and echocardiography was suggestive of severe left ventricular systolic dysfunction with EF of 20 %. He had a history of unsuccessful radiofrequency ablation 3 years ago, after which he was started on oral ivabradine, to which the tachycardia responded.

Using CARTO 3D EAM, the tachycardia was mapped to the tip of the right atrial appendage, where the earliest signal was 35 msec earlier than the surface P waves (Fig. 3B). Ablation at that site using an irrigated catheter resulted in immediate termination of the tachycardia (Supplementary Fig. 3). However, the patient had a recurrence of the tachycardia the next day (Supplementary Fig. 4), and he was restarted on ivabradine. Tachycardia responded to ivabradine (Supplementary Fig. 5), and the ejection fraction improved to normal on follow up, and there has been no recurrence of tachycardia on oral ivabradine treatment at a follow-up of 10 months. The average power used was 30 Watts with an RF duration of 3 minutes. The total procedure time was 120 minutes, and the fluoroscopy time was 9 minutes.

## 5 Case 4

**Fig. 4:**
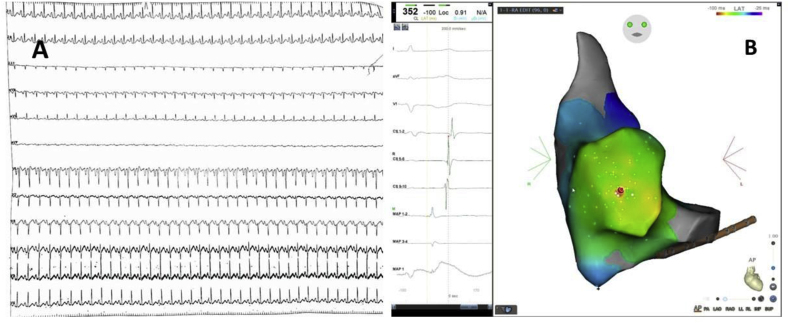
(A)ECG: EAT with negative P waves in V1 and positive P waves in inferior leads suggestive of origin from right atrial appendage. (B): 3D EAM showing unipolar and bipolar signals at the site of ablation.

A 38-year-old female presented with palpitations and dyspnoea on exertion for the past 2 months. Transthoracic echocardiography revealed LVEF of 40 % with global LV hypokinesia. ECG (Fig. 4A) was suggestive of EAT with origin from the right atrial appendage: negative P waves in V1 and positive P waves in inferior leads, and the patient was in incessant tachycardia on admission. Using CARTO 3D EAM, the tachycardia was mapped to the right atrial appendage, where the earliest activation was 48 msec earlier than the surface P waves (Fig. 4B). Tachycardia terminated after radiofrequency ablation using an irrigated catheter at that site. Post ablation, rhythm reverted to normal sinus rhythm with biphasic P wave in V1, and EF improved to normal on follow-up. The average power used was 30 Watts with an RF duration of 140 seconds. The total procedure time was 62 minutes, and the fluoroscopy time was 4 minutes. The patient continues to have sinus rhythm at a follow-up of 8 months. There were no complications in any of the cases.

## 6 Discussion

Ectopic atrial tachycardias originating from the atrial appendage are common in younger patients and are often incessant and associated with left ventricular dysfunction [1,2]. 3 patients in this series were younger than 25 years of age and had left ventricular systolic dysfunction on presentation, which improved on follow-up after successful management of the tachycardia. A male gender predominance has been described more characteristically for right atrial appendage (RAA) than left atrial appendage (LAA) tachycardias [1].

Right atrial appendage is a relatively rare site of origin for atrial tachycardias, accounting for only 8 % of cases of atrial tachycardias in a study [1]. Ectopic atrial tachycardia originating from the distal portion of the left atrial appendage is even rarer, accounting for 2.1 % reported cases of AT in a study [3]. Pulmonary vein ostia and atrial appendages are common sites of incessant atrial tachycardia [4].

Radiofrequency ablation is the treatment of choice for atrial appendage tachycardias with a high success rate, with a reported success rate of 92.8 % for left atrial appendage and 100 % for right atrial appendage in different studies [1,3]. Because of their incessant nature, these tachycardias are commonly present spontaneously during ablation and induction with atrial burst pacing or isoprenaline is rarely needed.

In appendage tachycardias, Bipolar EGMs can be used to find the ablation target and unipolar EGM is used to fine-tune the target. The distal unipolar EGM and the distal bipolar EGM of the mapping catheter should begin simultaneously, and there should be no other site recording earlier near-field or far-field activation. Bipolar EGMs are more reliable due to criss cross thick pectinate muscles and preferential conduction.

The trabeculated nature of atrial appendages makes catheter manipulation and adequate power delivery difficult. Thus, irrigation catheters are essential because of high interface temperatures owing to minimal cooling caused by blood flow in the atrial appendage. An irrigated tip catheter should deliver radiofrequency energy with a temperature limited to 43–45 °C. Radiofrequency energy should be started at 20 W and gradually increased up to 30–35 W. Lower temperature and power should be used in the apex of the appendage as compared to the base and the mid segment [3]. Care must be taken as perforation of the thin-walled atrial appendage is a potential complication.

For rare cases of atrial appendage tachycardia unresponsive to radiofrequency ablation, surgical excision of the atrial appendage is reasonable, given the incessant nature of the tachycardia and high prevalence of tachycardia-associated cardiomyopathy in these patients [5]. Also, there are case reports of Ectopic atrial tachycardia originating from right atrial appendage aneurysms in children, which are often refractory to medications and radiofrequency ablation [6]. Pre-procedure imaging by computed tomography (CT) can be used to look for aneurysms of the atrial appendage, and surgery can be considered in such cases. Minimally invasive appendectomy and epicardial exclusion of the appendage with a minimally invasive occlusion device have also been reported. Open surgical appendage ligation remains a last resort option for cases refractory to all other techniques [7].

Another option for patients with atrial tachycardias from the atrial appendage is oral ivabradine. In a study, it was seen that ivabradine-sensitive atrial tachycardia constitutes 64 % of incessant atrial tachycardias in patients without structural heart disease, and incessant atrial tachycardia originating in the atrial appendages is more likely to respond to ivabradine than that arising from other atrial sites [8]. As ivabradine is efficacious without any deleterious hemodynamic effects, a trial of ivabradine (10 mg single dose) should be considered in all patients with incessant atrial tachycardia and structurally normal hearts. In responders, ivabradine may be continued as a bridge to ablation or as destination therapy. Careful evaluation of the P wave morphology on the electrocardiogram is the key to early diagnosis and treatment, as the tachycardia might be misinterpreted as sinus tachycardia due to associated left ventricular dysfunction if P waves are not seen carefully [9,10].

Epicardial ablation or cryoablation are other options in some refractory cases. Cryoablation reduces the risk of perforation and tamponade in the thin-walled atrial appendage. Since the appendage is an area of low blood flow, RF ablation is relatively less effective and may cause char formation or steam pop on the catheter tip. In contrast to RF ablation, where the catheter remains mobile during ablation, solid tip cryoablation provides a freeze-mediated catheter adhesion to the target tissue, providing greater stability [11].

## 7 Conclusion

Ectopic atrial tachycardia from the atrial appendage is a common cause of incessant tachycardia and tachycardia-induced cardiomyopathy, which can be easily localized using P wave morphology changes on surface 12-lead electrocardiogram. Treatment options include radiofrequency ablation, cryoablation, oral ivabradine and, in some cases, atrial appendectomy.

## Ethical Statement

1) This material is the authors' own original work, which has not been previously published elsewhere.
2) The paper is not currently being considered for publication elsewhere.
3) The paper reflects the authors' own research and analysis in a truthful and complete manner.
4) The paper properly credits the meaningful contributions of co-authors and co-researchers.
5) The results are appropriately placed in the context of prior and existing research.
6) All sources used are properly disclosed (correct citation). Literally copying of text must be indicated as such by using quotation marks and giving proper reference.
7) All authors have been personally and actively involved in substantial work leading to the paper, and will take public responsibility for its content.

The violation of the Ethical Statement rules may result in severe consequences.

## Funding sources

This research did not receive any specific grant from funding agencies in the public, commercial, or not-for-profit sectors.

## Declaration of competing interest

The authors declare that they have no known competing financial interests or personal relationships that could have appeared to influence the work reported in this paper.
