# Supplementary material for: Tachycardia induced cardiomyopathy due to ectopic atrial tachycardia originating from the atrial appendage: A case series and review of literature
Source: Indian Pacing Electrophysiol J. 2025 Mar 6;25(2):112–7. doi: 10.1016/j.ipej.2025.03.001 (PMC12137971; doi:10.1016/j.ipej.2025.03.001)
Supplement: Multimedia component 1 [file mmc1.docx]

**SUPPLEMENTARY APPENDIX**


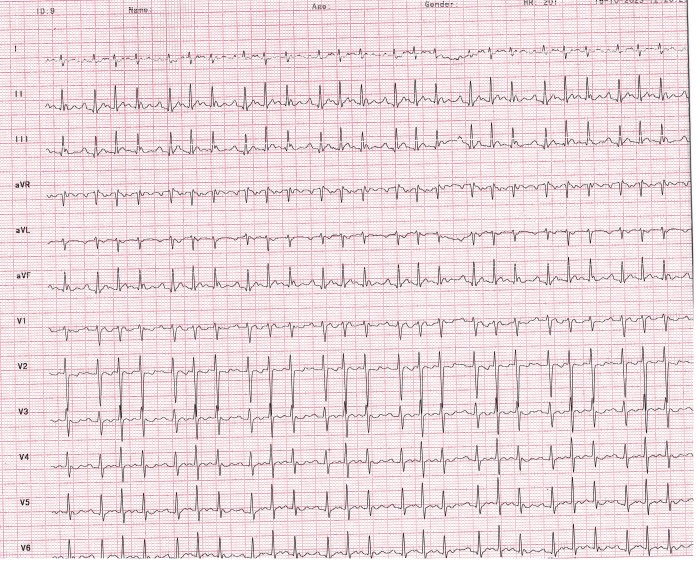


**SUPPLEMENTARY FIG 1:** ECG after giving adenosine: unmasking of the P waves


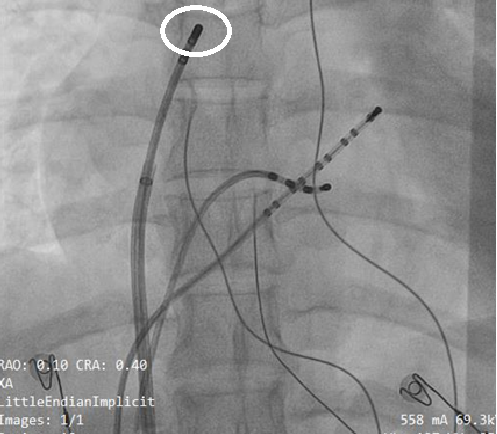


**SUPPLEMENTARY FIG 2:** Using SL 0 long sheath for support, ablation was done using irrigation ablation catheter at base of right atrial appendage


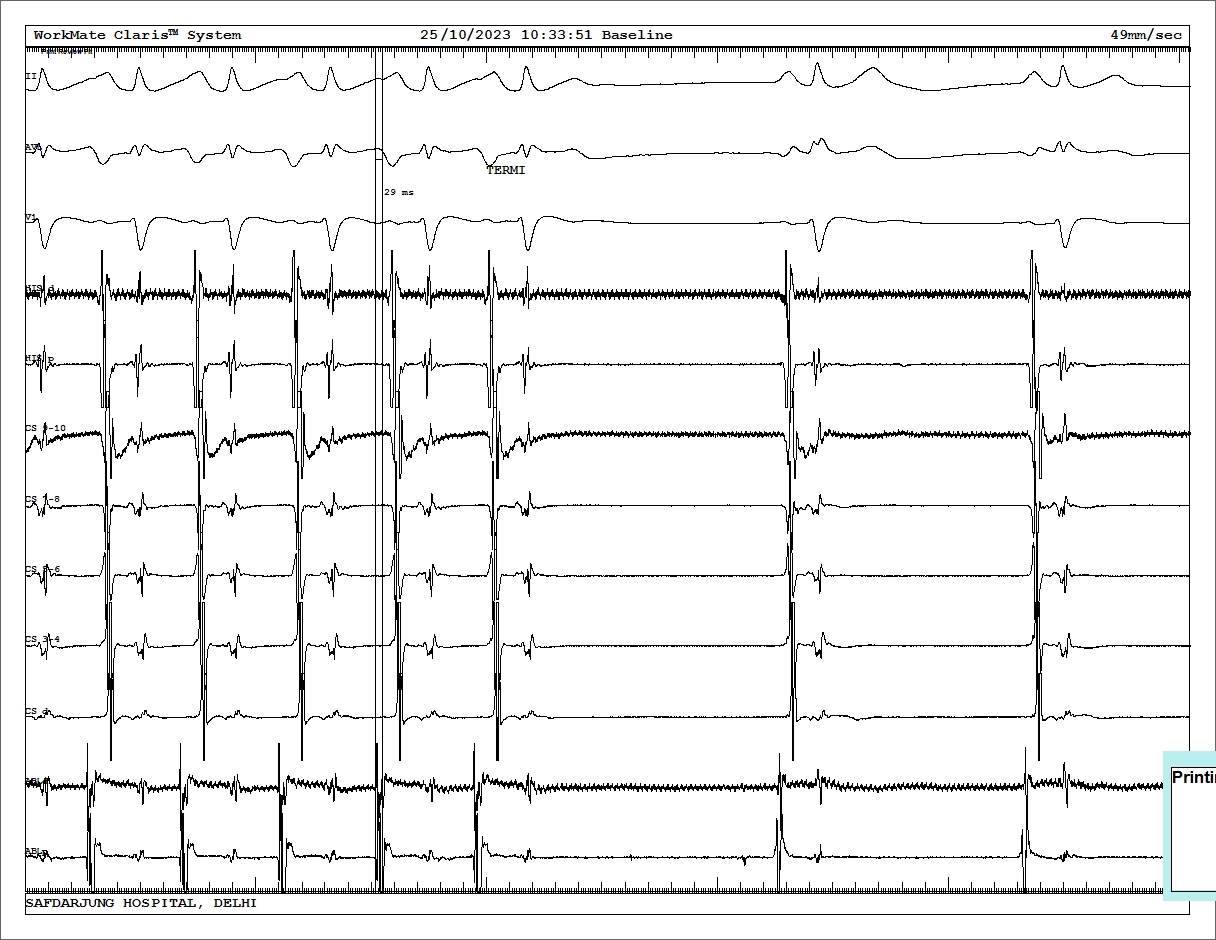


**SUPPLEMENTARY FIG 3**: TERMINATION OF TACHYCARDIA


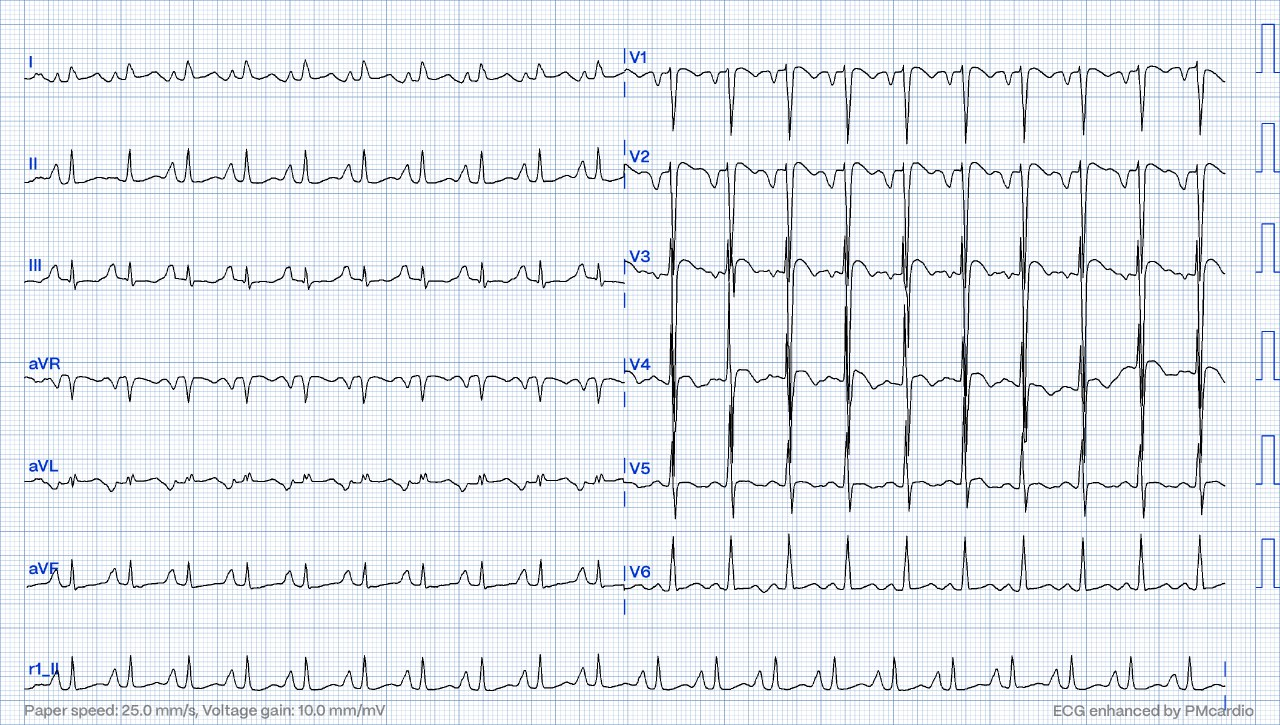


**SUPPLEMENTARY FIG 4**: ECG showing recurrence of the tachycardia after ablatiom


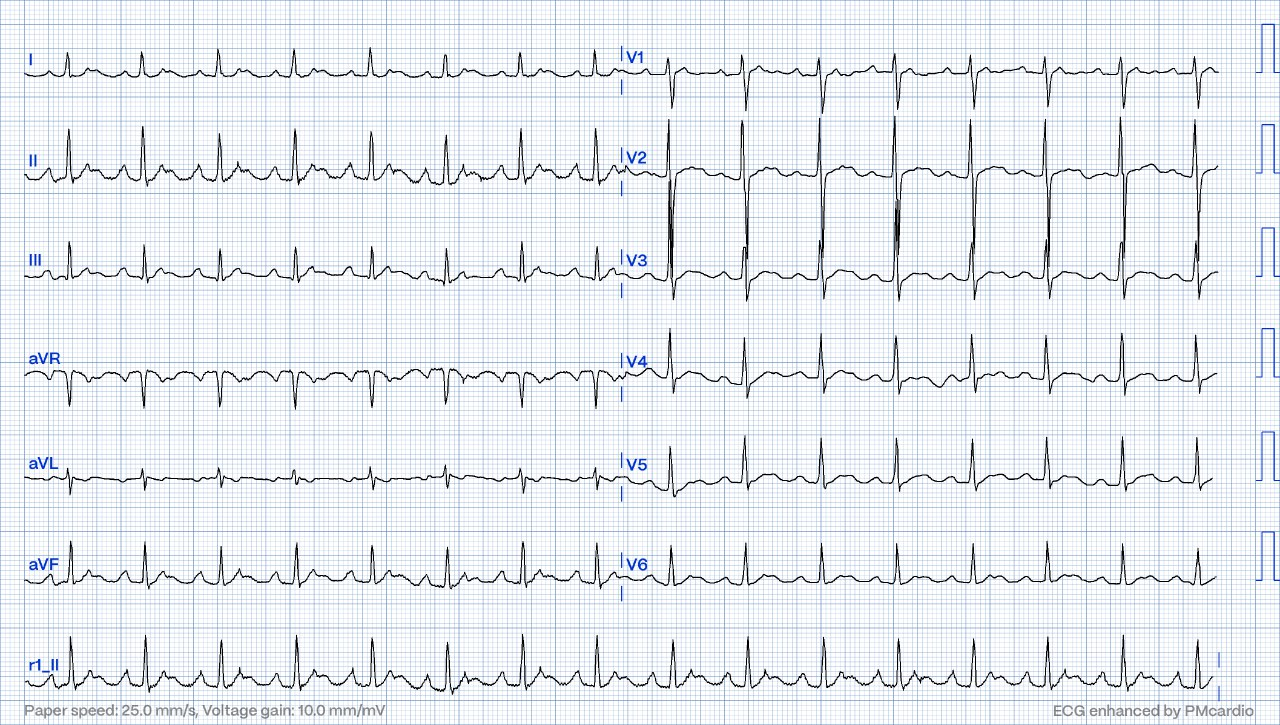


**SUPPLEMENTARY FIG 5**: ECG in normal sinus rhythm: on oral ivabradine
